# Predicting Delayed Postoperative Length of Stay Following Robotic Kidney Transplantation: Development and Simulation of Perioperative Risk Factors

**DOI:** 10.3390/medicina60081255

**Published:** 2024-08-01

**Authors:** Sang-Wook Lee, Kyoung-Sun Kim, Sung-Hoon Kim, Ji-Yeon Sim

**Affiliations:** Brain Korea 21 Project, Department of Anesthesiology and Pain Medicine, Asan Medical Center, University of Ulsan College of Medicine, Seoul 05505, Republic of Korea; sangwooklee@amc.seoul.kr (S.-W.L.); kyoungsun.kim@amc.seoul.kr (K.-S.K.); shkimans@amc.seoul.kr (S.-H.K.)

**Keywords:** robot-assisted kidney transplantation, hospital stay, delayed discharge, risk score, prediction, postoperative outcome

## Abstract

*Background and Objective:* Early discharge following robot-assisted kidney transplantation (RAKT) is a cost-effective strategy for reducing healthcare expenses while maintaining favorable short- and long-term prognoses. This study aims to identify predictors of postoperative delayed discharge in RAKT patients and develop a predictive model to enhance clinical outcomes. *Materials and Methods:* This retrospective study included 146 patients aged 18 years and older who underwent RAKT at a single tertiary medical center from August 2020 to January 2024. Data were collected on demographics, comorbidities, social and medical histories, preoperative labs, surgical information, intraoperative data, and postoperative outcomes. The primary outcome was delayed postoperative discharge (length of hospital stay > 7 days). Risk factors for delayed discharge were identified through univariate and multivariate regression analyses, leading to the development of a risk scoring system, the effectiveness of which was evaluated through receiver operating characteristic curve analysis. *Results:* 110 patients (74.8%) were discharged within 7 days post-transplant, while 36 (24.7%) remained hospitalized for 8 days or longer. Univariate and multivariate logistic regression analyses identified ABO incompatibility, BUN levels, anesthesia time, and vasodilator use as risk factors for delayed discharge. The RAKT score, incorporating these factors, demonstrated a predictive performance with a C-statistic of 0.789. *Conclusions:* This study identified risk factors for delayed discharge after RAKT and developed a promising risk scoring system for real-world clinical application, potentially improving postoperative outcome stratification in RAKT recipients.

## 1. Introduction

Kidney transplantation is the preferred treatment for patients with end-stage renal disease, offering a potential enhancement in quality of life and prolongation of survival [1]. Initially performed in 1954 [2], kidney transplantation has become the most commonly practiced surgical intervention for end-stage renal disease, traditionally employing the open surgical approach. However, recent advancements have seen the integration of robotic systems into transplant surgeries, signifying a notable shift in surgical techniques. The inaugural endeavor in robot-assisted kidney transplantation (RAKT) was undertaken by Giulianotti et al. in 2010 [3] and has since been adopted across numerous centers worldwide. This surge in popularity is attributable to its advantages, including reduced incision sizes and a decreased incidence of postoperative surgical site infections, presenting a compelling case for its continued integration into transplant surgery practices [4,5]. Early discharge following surgical procedures emerges as a highly cost-effective strategy for curbing healthcare expenses. Likewise, early discharge after kidney transplantation is considered a viable approach to optimize the efficient use of healthcare resources. This strategy holds potential for reducing healthcare costs without markedly impacting short- and long-term postoperative outcomes. A prior study, analyzing extensive data from a national database, explored the effects of early discharge on outcomes following kidney transplantation and found it to be cost-effective, with no discernable correlation to reduced post-transplant survival rates or elevated 90-day readmission rates [6]. Previous studies have shown that RAKT is associated with fewer surgical site infections, reduced postoperative pain, and shorter hospital stays compared with open kidney transplantation (OKT), owing to the smaller incision site [4,7,8,9,10,11]. However, RAKT also presents challenges such as longer operative times, extended cold ischemia times, and difficulties with the intraoperative steep Trendelenburg position and pneumoperitoneum, potentially adversely affecting postoperative outcomes [12]. Another challenging aspect of RAKT is the lack of established intraoperative fluid management guidelines. Therefore, it is necessary to follow the existing fluid management guidelines for OKT while actively utilizing new monitoring metrics such as stroke volume variation, which may better reflect intraoperative fluid status than central venous pressure (CVP), considering the unique features of RAKT, such as the steep Trendelenburg position and pneumoperitoneum [13]. To date, there has been a paucity of research exploring the perioperative risk factors, including intraoperative parameters such as blood pressure and heart rate, that contribute to delayed discharge in RAKT compared with OKT. Consequently, this study aims to identify predictors of postoperative delayed discharge in individuals undergoing RAKT and to develop a predictive model for assessing the risk of delayed discharge after RAKT. This model is intended to aid clinicians in enhancing patient outcomes through informed decision-making in clinical practice.

## 2. Methods

### 2.1. Study Design

This study enrolled adult patients aged 18 years and above who underwent RAKT at a tertiary medical center from August 2020 to January 2024. Exclusion criteria included pediatric patients younger than 18 years and cases necessitating intraoperative conversion from robotic to open surgery. The study adhered to the guidelines of the Declaration of Helsinki and received approval from the Institutional Review Board (IRB) of the tertiary medical center. Data for this study were retrospectively collected and analyzed from the institution’s electronic medical records. Consequently, the IRB granted a waiver for the requirement for informed consent.

### 2.2. Data Variables

This study comprehensively analyzed various data variables, encompassing demographic data, comorbid conditions, social and medical histories, preoperative laboratory results, history of preoperative medication, surgical details, intraoperative data, donor characteristics, and postoperative outcomes. Demographic data included essential parameters such as age, sex, weight, height, and body mass index (BMI) of the participants. Patient comorbidity data encompass various conditions, such as hypertension (HTN), diabetes mellitus (DM), coronary artery disease (CAD), congestive heart failure (CHF), history of percutaneous coronary intervention (PCI) or coronary artery bypass grafting (CABG), and cerebrovascular accidents (CVA), among others. Social and medical history comprises details on smoking and alcohol consumption, as well as the patient’s preoperative hemodialysis status, including the duration of such treatment. Variables for preoperative laboratory tests encompass hemoglobin (Hb), creatinine, blood urea nitrogen (BUN), albumin, sodium, potassium, and estimated glomerular filtration rate (eGFR). The preoperative medication history encompasses medications such as angiotensin-converting enzyme inhibitors (ACE-Is), angiotensin II receptor blockers (ARBs), beta-blockers, and calcium channel blockers (CCBs). Surgical information encompasses details such as the duration of the surgery, anesthesia time, cold ischemia time (CIT), warm ischemia time (WIT), and rewarming time (RWT). Intraoperative data comprise variables such as operation time, cold ischemia time, rewarming time, administration of inotropes or vasodilators, diuretic dose, the volume of fluids administered, necessity and extent of blood transfusions, urine output, blood loss, heart rate, blood pressure, and CVP. Data pertaining to the donor include sex, age, BMI, and medical history, regarding HTN or DM. Postoperative outcome data encompass variables such as the patient’s postoperative dialysis status, serum creatinine levels after surgery, postoperative mortality, postoperative intensive care unit (ICU) admission rate, and the postoperative length of hospital stay. 

### 2.3. Anesthesia and Perioperative Management in RAKT

All participants received preoperative administration of immunosuppressive medications following the institution’s protocol, which included calcineurin inhibitors, methylprednisolone, and mycophenolate mofetil, administered 2 or 7 days before the surgical procedure. Supplementary immunosuppressants such as anti-thymocyte globulin or basiliximab were provided intraoperatively. Pre-anesthesia monitoring comprised electrocardiography, non-invasive blood pressure measurement, pulse oximetry, neuromuscular monitoring, and bispectral index (BIS) assessments to ensure comprehensive monitoring. Anesthetic induction was achieved with propofol or remimazolam, followed by rocuronium for muscle relaxation, facilitating endotracheal intubation. Intravenous anesthesia maintenance involved propofol and remifentanil. Post-induction, an arterial catheter was inserted into the radial artery using the sterile technique for continuous blood pressure monitoring and arterial blood gas analyses. A central venous catheter was also placed for central venous pressure monitoring and the administration of fluids and medications during the operation. Normothermia was preserved through the use of warming devices such as fans and heated sheets. At the initiation of graft kidney vascularization, 20% mannitol was administered to induce diuresis, supplemented by furosemide administration before and after the reperfusion process to enhance renal function and maintain fluid balance. According to previous studies and our center’s protocol, intraoperative blood pressure was maintained using vasopressors or volume replacement to achieve a systolic blood pressure of 150 mm Hg from the moment of reperfusion onwards [14,15]. If intraoperative blood pressure was lower than the predetermined threshold at reperfusion, 5 to 10 mg of ephedrine or 2.5 to 5 mcg of norepinephrine was administered. Nitroglycerin (NTG) was employed to manage episodes of significant intraoperative hypertension, thereby optimizing hemodynamic stability throughout the surgical procedure. For the successful execution of robotic surgical procedures, patients were placed in a Trendelenburg position, specifically angled between 20 to 30 degrees, to facilitate optimal surgical access and visibility. Additionally, a pneumoperitoneum was established and maintained at a pressure of approximately 10 mmHg throughout the procedure to ensure enhanced intraoperative visualization. Although some patients needed postoperative ICU care due to inadequate spontaneous breathing recovery, unstable hemodynamic status, and surgical complications, the majority were discharged from the general ward without major complications after their postoperative recovery period.

### 2.4. Primary and Secondary Outcomes

In our study, the primary outcome was postoperative delayed discharge (postoperative hospital length of stay > 7 days). Postoperative delayed discharge was defined as discharge occurring more than 7 days after transplant surgery. Secondary outcomes encompassed delayed graft failure, the incidence of major adverse cardiac events (MACE) following surgery, postoperative patient survival rates, instances of graft rejection, readmission within 90 days, and specifically, postoperative intensive care unit (ICU) admission rate.

### 2.5. Statistical Analysis

Continuous variables were compared for differences using Student’s *t*-test or the Mann–Whitney U test, as appropriate. Categorical variables were compared using the chi-squared test. To build a model predicting postoperative delayed discharge development, we included variables with *p*-values less than 0.1 from univariate logistic regression analysis as candidate variables and fitted the final multivariable model using stepwise selection. When selecting variables, we examine their variance inflation factor (VIF) values, and if a variable has a VIF value of 10 or more, we determine that there was multicollinearity and exclude it from the analysis. A new predictive scoring system was derived from the beta regression coefficients of identified risk factors, obtained through multivariable logistic regression analysis. The discriminative capability of the predictive scoring system was assessed using receiver operating characteristic (ROC) curve analysis, while its calibration performance was evaluated using the Hosmer–Lemeshow goodness-of-fit test. All statistical analyses were conducted utilizing the R statistical software (version 4.3.3, R Foundation for Statistical Computing, Vienna, Austria). The level of significance across all statistical analyses was set at a *p*-value of less than 0.05.

## 3. Results

### 3.1. Study Patient Characteristics

In this study, among the 146 recipients of robot-assisted kidney transplantation, 110 (75.3%) were discharged within 7 days post-transplant, while the remaining 36 (24.7%) were discharged after 8 or more days post-transplant. Comparison between the early discharge group (discharged within 7 days) and the delayed discharge group (discharged after 8 days or more) revealed that the early discharge group had a younger average age (43.9 vs. 45.4 years, respectively; *p*-value = 0.489) and a higher proportion of female patients (44.5% vs. 38.9%, respectively). However, the differences in age and sex were not statistically significant (*p* = 0.489 and 0.688, respectively) (Table 1). 

Values are presented as mean ± standard deviation or number (percentage). BMI, body mass index; HTN, hypertension; DM, diabetes mellitus; mFI-5, modified 5-item frailty index; PCI, percutaneous coronary intervention; CABG, coronary artery bypass graft; CRF, chronic renal failure; FSGS, focal segmental glomerulosclerosis; PCKD, polycystic kidney disease; ACE, angiotensin-converting enzyme; ARB, angiotensin receptor blocker; CCB, calcium channel blocker, BUN, blood urea nitrogen; eGFR, estimated glomerular filtration rate; RBC, red blood cell; HR, heart rate; SD, standard deviation; SBP, systolic blood pressure; DBP, diastolic blood pressure; MBP, mean blood pressure; CVP, central venous pressure; DGF, delayed graft failure; MACE, major adverse cardiovascular event.

The analysis showed a statistically significant difference in BMI between the groups, with the early discharge group presenting a lower mean BMI of 22.7 ± 4.6 compared with 24.4 ± 5.0 in the delayed discharge group (*p* = 0.050). Compared with the early discharge group, the delayed discharge group exhibited a higher rate of ABO incompatibility (38.9% vs. 21.8%, *p* = 0.071) and a greater proportion of patients receiving preoperative hemodialysis (91.7% vs. 72.2%, *p* = 0.03). 

Preoperative laboratory data revealed that the delayed discharge group had significantly lower BUN and potassium levels compared with the early discharge group (*p* = 0.004 and 0.005, respectively). Furthermore, intraoperative data indicated that the delayed group experienced longer operative times (*p* = 0.009), higher rates of intraoperative vasodilator use (*p* = 0.023), and higher doses of diuretics (*p* = 0.014), and they received greater volumes of crystalloid fluids (*p* = 0.009). 

Although no intraoperative blood pressure parameter showed a statistically significant difference between the two groups, the mean variations of systolic blood pressure (mean of ΔSBP, *p* = 0.056), diastolic blood pressure (mean of ΔDBP, *p* = 0.055), and mean blood pressure (mean of ΔMBP, *p* = 0.057) approached statistical significance, suggesting notable differences in intraoperative blood pressure variability.

Among postoperative complications, three patients (8.3%) in the delayed group developed delayed graft failure (DGF), compared to none in the early group (*p* = 0.017).

### 3.2. Risk Factors Influencing Delayed Discharge after RAKT

To analyze risk factors for delayed postoperative discharge in patients undergoing RAKT, we initially conducted univariate and multivariate logistic regression analyses. Variables with *p*-values of less than 0.1 in the univariate analysis were selected as candidates for multivariate regression analysis. Through stepwise selection, the final four risk factors identified were ABO incompatibility, BUN levels, operation time, and vasodilator use (Table 2). Higher BUN levels were inversely associated with delayed discharge (AOR: 0.97; 0.95–0.99; *p* = 0.005), ABO incompatibility (AOR: 2.20; 0.97–4.96; *p*-value = 0.059), extended operation time (AOR: 1.01; 1.00–1.01, *p* = 0.007), and vasodilator use (AOR: 2.41; 1.05–5.55, *p* = 0.038), and increased the probability of a postoperative stay exceeding 8 days. 

### 3.3. RAKT Score

The new risk scoring system, termed the RAKT score, is detailed in Table S1, including a breakdown of the risk scores corresponding to the values of the four risk factors. These individual risk scores are then aggregated to calculate the final RAKT total score. The RAKT score is determined using the β coefficient values from the multivariate logistic regression of the four selected risk factors. The reference score was set at 10 points for a 500-unit increase in Anesthesia time (β_ref_ = 0.008), and each score determined proportionally the magnitude of the β coefficient (e.g., score of ABOi = β_ABOi_/(β_ref_ × 500) × 10). The formula provided below outlines the calculation of the RAKT score based on the values of each risk factor.

$$\mathrm{RAKT}\;\mathrm{score}=2.0\;\times \;\mathrm{ABOi}+\frac{(130-BUN)\times 8.0}{110}+\frac{\left(Anesthesia\;time-250\right)\times 10}{500}\;+\;2.2\;\times \;\mathrm{Vasodilator}$$

**Figure 1:**
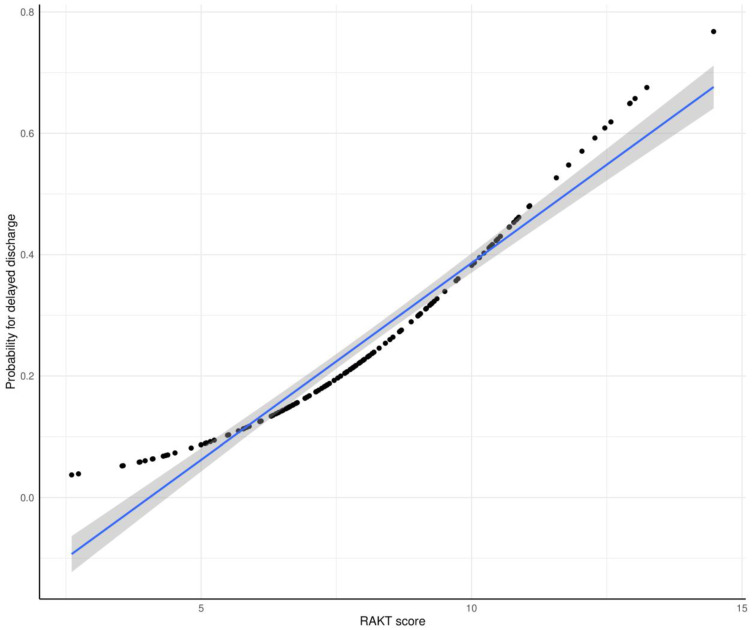
Predictive performance of the RAKT score for delayed discharge in patients undergoing robot-assisted kidney transplantation (postoperative hospital stay > 7 days). The black dots show the distribution of the actual values of the probability of delayed discharge by RAKT score. The blue solid line represents the linear regression line of this distribution, and the gray area indicates the confidence interval of the regression line. RAKT, robot-assisted kidney transplantation.

**Figure 2:**
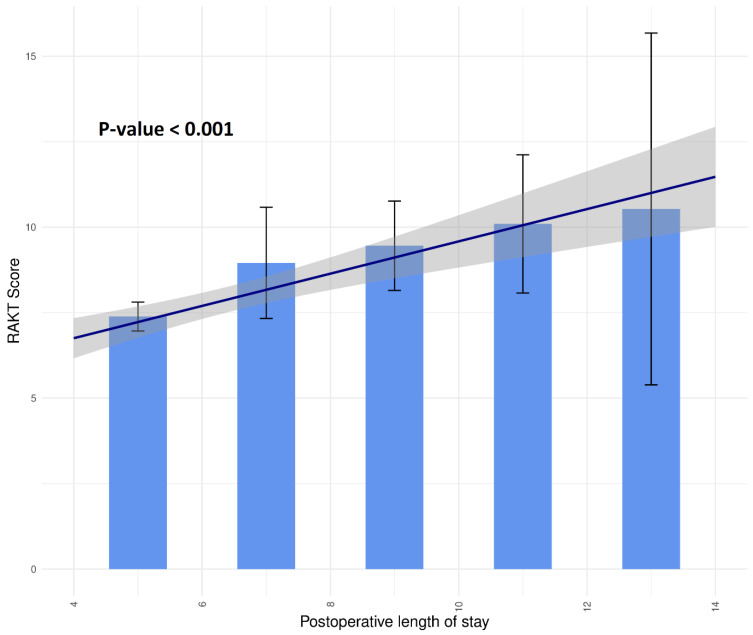
Delayed discharge probability distribution by RAKT score. The *p*-value indicates the significance level for the trend regression line. The blue solid line represents the trend line for the distribution of RAKT scores in relation to the postoperative length of stay, with the gray area indicating the confidence interval of this trend line. The black solid bar lines depict the confidence intervals of the corresponding RAKT score distribution for each value of postoperative length of stay. RAKT, robot-assisted kidney transplantation.

Categorical variables, such as ABO incompatibility (ABOi) and vasodilator use, were assigned a value of 1 to indicate presence and 0 for absence. Analysis of the RAKT score nomogram reveals an inverse relationship with BUN levels and direct associations with ABOi, longer operation time, and vasodilator use (Figure S1). The RAKT score ranges from 0 to 16, with higher scores correlating with an increased likelihood of delayed discharge (Figure 1). Furthermore, Figure 2 depicts the distribution of RAKT scores in relation to postoperative hospital stay duration, demonstrating a rise in RAKT scores as hospitalization post-surgery extends (*p*-value < 0.001).

### 3.4. Predictive Performance of RAKT Score for Delayed Postoperative Discharge

**Figure 3:**
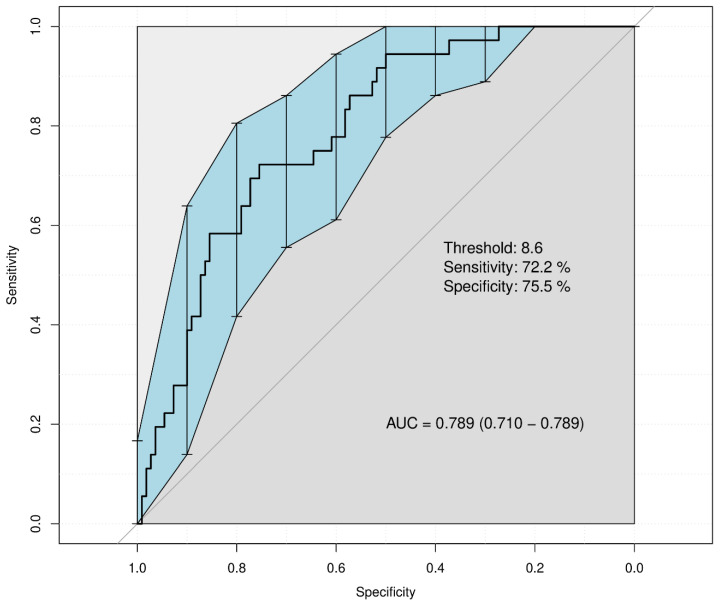
Distribution of RAKT scores by postoperative hospital length of stay in patients undergoing robot-assisted kidney transplantation. The black vertical solid lines indicate the confidence intervals for each point on the ROC curve, and the blue area connects these intervals to illustrate the confidence interval for the overall ROC curve. RAKT, robot-assisted kidney transplantation; AUC, area under the curve.

To evaluate the predictive performance of the RAKT score-based delayed discharge risk prediction model, ROC curve analysis and calibration curve evaluation were conducted. The area under the ROC curve (AUC) was 0.789 (95% CI: 0.710–0.789), indicating a strong predictive capability (Figure 3). Figure S2 illustrates the calibration of the risk prediction model, demonstrating a close alignment between predicted probabilities and actual outcomes.

### 3.5. Clinical Outcomes Associated with RAKT Score Distribution

The ROC analysis determined an optimal cutoff value of 8.6 for predicting delayed discharge with the RAKT score. At this threshold, the sensitivity was 72.2% and the specificity was 75.5%. Utilizing this cutoff, RAKT scores were categorized into three groups for further analysis: a low RAKT score group (<8), a medium RAKT score group (8–9, inclusive of the cutoff value), and a high RAKT score group (>9). Clinical outcomes for these groups are detailed and compared in Table 3. A statistically significant difference was observed in the postoperative length of stay among the three groups, with the high RAKT score group experiencing a longer stay (11.9 days) compared with the low RAKT score group (6.2 days) (*p* < 0.001). Additionally, clear distinctions were noted among the groups in terms of eGFR on postoperative day 7 (*p* = 0.009), eGFR at discharge (*p* = 0.015), creatinine levels at discharge (*p* = 0.024), and postoperative ICU admission (*p* = 0.003). However, although no statistically significant differences were observed in 90-day readmission rates among the three groups (*p* = 0.372), the limited number of events for outcomes, such as DGF (*p*-value = 0.318), MACE (*p* = 0.350), and organ rejection (*p* = 0.331), posed challenges in determining their statistical significance.

## 4. Discussion

Early discharge following surgery is increasingly recognized as a beneficial strategy for optimizing healthcare resource utilization [16,17,18,19]. A previous study involving 61,798 kidney transplant patients demonstrated that early discharge was not only cost-effective but also did not correlate with a reduction in post-transplant survival rates or an increase in 90-day readmission rates [6]. Consequently, facilitating early discharge after kidney transplantation emerges as a promising approach to reducing healthcare costs compromising postoperative patient outcomes [20]. Delayed discharge after kidney transplantation may be closely associated with patients’ underlying comorbidities and the onset of postoperative complications. Such complications, including the need for postoperative dialysis or infection development, can lead to extended postoperative hospital stays [21]. Evaluating the risk of delayed discharge after kidney transplantation is crucial for anticipating postoperative outcomes. Proactively identifying risk factors for prolonged postoperative hospitalization and formulating a predictive system based on these factors constitute an effective strategy for optimizing medical resource allocation. This enables the pre-surgical screening of at-risk patients, facilitating the strategic allocation of resources towards those at higher risk. Notably, patients undergoing RAKT generally experience shorter post-surgical hospitalizations compared with those undergoing OKT. This disparity can be attributed to factors such as smaller incisions and reduced complication rates in RAKT. Consequently, it is plausible that the risk factors influencing discharge timing may differ between RAKT and OKT cohorts [4,8,9,10,11,22,23]. Diverging from previous studies focused on OKT, our research identifies unique risk factors for delayed discharge among RAKT patients and develops a predictive scoring system based on these identified risk factors [6].

In our study, we identified ABO incompatibility, decreased BUN, prolonged anesthesia duration, and the intraoperative use of vasodilators as primary risk factors for delayed postoperative discharge in patients undergoing RAKT. Despite recent advancements in desensitization protocols and optimization strategies for ABO-incompatible kidney transplantation, ABO-incompatible kidney transplants (ABOi KTs) carry a higher risk of postoperative infections, organ rejection, and bleeding compared with ABO-compatible kidney transplants (ABOc KTs) [24]. Therefore, ABO incompatibility emerges as a significant contributor to delayed hospital discharge after kidney transplantation. The prolonged duration of surgery typically indicates increased procedural complexity, which in turn is associated with a heightened risk of postoperative complications [25]. Within the realm of transplant surgery, extended operative times also lead to prolonged warm ischemia time before reperfusion, potentially impacting the function of the transplanted kidney postoperatively [26]. However, our study found that the duration of rewarming alone did not constitute an independent risk factor for delayed discharge. In our investigation, the administration of intraoperative blood pressure-lowering medications emerged as a potential risk factor for prolonged hospital stay. Previous research has rarely explored the relationship between the use of these medications during surgery and postoperative recovery. Given that the need for such medications indicates the presence of intraoperative hypertension, further research is warranted to assess the impact of deliberate intraoperative hypertension management on postoperative outcomes.

Although a previous study highlighted that maintaining systolic blood pressure above 150 mmHg at the time of reperfusion was associated with early stabilization of graft function in kidney transplantation [15], most of the existing research has overlooked the effects of intraoperative vital sign metrics, such as blood pressure and heart rate, on the outcomes of patients undergoing kidney transplantation. This study aimed to analyze how intraoperative blood pressure, heart rate, and other vital signs influence the incidence of delayed discharge postoperatively. We computed the mean and SD of all intraoperative blood pressure and heart rate fluctuations to provide a statistical overview. Additionally, we calculated the differences in blood pressure and heart rate to assess variability indicators. These measures, along with the calculated mean and SD values, were then analyzed. In univariate regression analysis, the mean and SD of delta SBP, and the mean of delta MBP, significantly influenced postoperative delayed discharge (*p* < 0.05). However, this significance was not observed when adjusting for other significant variables in the multivariate regression analysis. Additionally, although the scope of this study was confined to assessing the influence of vital parameters, such as intraoperative blood pressure and pulse rate, on delayed postoperative discharge, future large-scale analyses are expected to leverage important data to explore the relationship between these intraoperative metrics and various postoperative outcomes.

Our study stands as one of the few investigations into the outcomes of patients undergoing RAKT, an area scarcely covered in the literature. The substantial dataset we have amassed over a single period is particularly noteworthy, enabling us to comprehensively analyze the short-term outcomes of RAKT patients. Furthermore, our study identifies the risk factors associated with delayed discharge and introduces a novel risk-scoring system, contributing significantly to the field by offering insights and tools that were previously unavailable.

However, despite the clinical relevance of our findings, our study is not without its limitations. First, the data were collected over a single period from a single institution, prompting concerns about the generalizability of our risk prediction system to other settings. It is imperative to validate the applicability of our system with data from diverse institutions to confirm its clinical utility. Additionally, the incidence of postoperative complications such as delayed graft failure, organ rejection, and mortality was significantly lower in our cohort, limiting our ability to identify risk factors for these severe outcomes due to the small sample size. Although data from a significant number of patients who underwent RAKT have been collected in a short timeframe, expanding this analysis to include larger datasets from multiple centers is crucial. Future research should focus on verifying the effectiveness of the proposed risk prediction scoring system in diverse clinical settings and its capability to forecast both short-term and long-term outcomes. Moreover, acquiring a more extensive dataset from various centers involving RAKT patients will allow for the refinement of the risk-scoring system. Such advancements will enhance its ability to effectively predict a wider range of clinical outcomes, thereby bolstering its utility in practical clinical scenarios. In addition, several recent studies have demonstrated that the enhanced recovery after surgery (ERAS) protocol reduces hospital stays and improves clinical outcomes in patients [27,28]. To summarize the typical ERAS protocol strategies after kidney transplantation, postoperative catheters should be removed as early as possible to reduce the risk of UTI, and prophylactic drain placement should be avoided. Additionally, early ambulation and resumption of diet after surgery are beneficial for early recovery. Education and counseling about medication regimens, such as immunosuppressants, are also critical components of the postoperative ERAS protocol strategy [29]. Although we were unable to apply the ERAS protocol due to the retrospective nature of our analysis of early-phase RAKT patients, further prospective studies utilizing the ERAS protocol in RAKT patients are warranted. This study only addressed clinical factors that affect hospital discharge. However, discharge can be influenced by several non-medical factors, such as socio-economic status, presence of a caregiver, proximity to the facility, and sometimes the logistics of discharge, such as a transfer to a highly skilled nursing facility. Future studies should consider these non-medical factors alongside the medical ones.

In conclusion, our examination of the study data revealed several factors associated with delayed discharge in patients receiving RAKT, notably including ABO incompatibility, BUN levels, anesthesia duration, and the intraoperative use of vasodilators. By incorporating these identified risk factors, we developed the RAKT score, a risk prediction scoring system for delayed discharge. This tool demonstrates efficacy in predicting postoperative outcomes, including delayed discharge, in patients undergoing RAKT. Additionally, this system demonstrates promise for real-world clinical application, serving as a valuable tool for risk stratification of postoperative outcomes in RAKT recipients. Our findings underscore the potential of the proposed scoring system to improve patient management and refine prognostic assessments in the post-transplantation setting.

## Figures and Tables

**Table 1:** Comparison of clinical characteristics in patients undergoing robotic kidney transplantation by post-transplant length of hospital stay.

	Total	Early (<8 Days)	Delayed (≥8 Days)	*p*-Value
Number of patients, *n* (%)	146	110 (75.3)	36 (24.7)	
Demographic data				
Age, years	44.3 ± 13.3	43.9 ± 13.2	45.4 ± 13.5	0.489
Female sex, *n* (%)	63 (43.2)	49 (44.5)	14 (38.9)	0.688
BMI, kg/m^2^	23.1 ± 4.7	22.7 ± 4.6	24.4 ± 5.0	0.050
Comorbidities				
HTN, *n* (%)	122 (84.1)	93 (85.3)	29 (80.6)	0.678
DM, *n* (%)	41 (28.1)	30 (27.3)	11 (30.6)	0.868
Coronary artery disease, *n* (%)	2 (1.4)	1 (0.9)	1 (2.8)	0.991
Congestive heart failure, *n* (%)	28 (19.2)	17 (15.5)	11 (30.6)	0.079
Cerebrovascular accidents, *n* (%)	1 (0.7)	0 (0.0)	1 (2.8)	0.562
mFI-5	1.1 ± 0.7	1.1 ± 0.7	1.3 ± 0.7	0.662
ABO incompatibility, *n* (%)	38 (26.0)	24 (21.8)	14 (38.9)	0.071
Preoperative hemodialysis, *n* (%)	111 (77.1)	78 (72.2)	33 (91.7)	0.030
Cause of CRF				0.571
HTN, *n* (%)	19 (13.0)	15 (13.6)	4 (11.1)	
DM, *n* (%)	31 (21.2)	21 (19.1)	10 (27.8)	
IgA, *n* (%)	34 (23.3)	23 (20.9)	11 (30.6)	
FSGS, *n* (%)	10 (6.8)	8 (7.3)	2 (5.6)	
PCKD, *n* (%)	27 (18.5)	22 (20.0)	5 (13.9)	
Unknown, *n* (%)	25 (17.1)	21 (19.1)	4 (11.1)	
Medication history				
ACE inhibitor or ARB, *n* (%)	57 (39.0)	45 (40.9)	12 (33.3)	0.541
Beta blocker, *n* (%)	60 (41.1)	41 (37.3)	19 (52.8)	0.148
CCB, *n* (%)	97 (66.4)	74 (67.3)	23 (63.9)	0.865
Preoperative laboratory data				
Hemoglobin, g/dL	10.6 ± 1.8	10.6 ± 1.7	10.3 ± 1.9	0.378
Creatinine, mg/dL	7.6 ± 2.8	7.8 ± 2.8	7.2 ± 2.8	0.406
BUN, mg/dL	60.7 ± 22.4	64.0 ± 23.4	50.5 ± 15.5	0.004
Albumin, g/dL	3.8 ± 0.7	3.8 ± 0.7	3.8 ± 0.5	0.213
Sodium, mmol/L	136.3 ± 3.4	136.0 ± 3.4	137.0 ± 3.4	0.229
Potassium, mmol/L	4.8 ± 0.9	4.9 ± 0.9	4.5 ± 0.8	0.005
eGFR, mL/min/1.73 m^2^	8.1 ± 4.1	7.7 ± 3.4	9.2 ± 5.5	0.175
Intraoperative data				
Operation time, min	371.0 ± 63.7	362.2 ± 52.6	397.7 ± 85.1	0.009
Cold ischemia time, min	106.1 ± 38.0	102.3 ± 33.5	118.4 ± 48.3	0.062
Rewarming time, min	58.8 ± 15.3	58.1 ± 12.3	61.2 ± 22.6	0.779
Inotropes, *n* (%)	9 (6.2)	5 (4.5)	4 (11.1)	0.307
Vasodilator, *n* (%)	31 (21.2)	18 (16.4)	13 (36.1)	0.023
Loop diuretic dose (Furosemide), mg	41.0 ± 9.2	39.9 ± 6.3	44.4 ± 14.4	0.014
Volume of Crystalloid, mL	1514.5 ± 675.2	1405.5 ± 560.7	1847.8 ± 870.9	0.009
Volume of Colloid, mL	466.6 ± 209.2	481.8 ± 206.8	420.2 ± 212.6	0.190
Transfusion (RBC), unit	0.2 ± 0.6	0.2 ± 0.5	0.3 ± 0.7	0.343
Urine output, mL	669.5 ± 537.2	701.8 ± 563.9	570.7 ± 437.9	0.220
Blood loss, mL	88.5 ± 56.3	84.9 ± 48.8	99.4 ± 74.6	0.227
HR (mean), bpm	68.6 ± 9.2	68.2 ± 8.9	69.9 ± 10.0	0.351
HR (SD), bpm	6.9 ± 2.7	6.9 ± 2.6	6.8 ± 3.0	0.426
ΔHR (mean), bpm	2.1 ± 1.0	2.1 ± 0.9	2.1 ± 1.1	0.531
ΔHR (SD), bpm	3.3 ± 1.9	3.4 ± 1.9	3.2 ± 1.9	0.310
SBP (mean), mmHg	137.0 ± 14.8	135.8 ± 13.5	140.6 ± 18.0	0.149
SBP (SD), mmHg	13.9 ± 4.1	13.8 ± 4.2	14.3 ± 3.7	0.533
ΔSBP (mean), mmHg	6.7 ± 2.3	6.4 ± 2.2	7.3 ± 2.6	0.056
ΔSBP (SD), mmHg	8.0 ± 3.5	7.6 ± 2.9	9.1 ± 4.7	0.113
DBP (mean), mmHg	72.1 ± 8.9	72.6 ± 8.6	70.5 ± 9.6	0.215
DBP (SD), mmHg	8.4 ± 3.2	8.2 ± 2.7	9.0 ± 4.4	0.305
ΔDBP (mean), mmHg	3.9 ± 1.5	3.7 ± 1.4	4.3 ± 1.8	0.055
ΔDBP (SD), mmHg	5.3 ± 4.2	5.0 ± 3.1	6.3 ± 6.6	0.241
MBP (mean), mmHg	93.7 ± 10.3	94.0 ± 10.1	92.9 ± 10.9	0.586
MBP (SD), mmHg	10.7 ± 3.4	10.4 ± 3.1	11.5 ± 4.2	0.116
ΔMBP (mean), mmHg	5.0 ± 1.9	4.8 ± 1.7	5.5 ± 2.2	0.057
ΔMBP (SD), mmHg	6.3 ± 4.2	5.9 ± 3.1	7.6 ± 6.4	0.080
CVP (mean), mmHg	10.6 ± 3.8	10.9 ± 3.7	9.8 ± 3.9	0.110
CVP (SD), mmHg	3.9 ± 1.8	4.0 ± 1.8	3.5 ± 1.8	0.088
Postoperative complications				
DGF, *n* (%)	3 (2.1)	0 (0)	3 (8.3)	0.017
Rejection, *n* (%)	3 (2.1)	2 (1.8)	1 (2.8)	1.000
MACE, *n* (%)	1 (0.7)	0 (0)	1 (2.8)	0.544

**Table 2:** Univariate and multivariate logistic regression analysis of various factors influencing delayed post-transplant length of hospital stay following robotic kidney transplantation.

Variables	Univariate		Multivariate	
	OR (95% CI)	*p*-Value	OR (95% CI)	*p*-Value
Age, years	1.01 (0.98–1.04)	0.549		
Female sex	0.79 (0.37–1.71)	0.552		
BMI	1.07 (0.99–1.16)	0.074		
Preoperative hemodialysis	4.23 (1.21–14.8)	0.024		
HTN	0.71 (0.27–1.90)	0.499		
DM	1.17 (0.52–2.68)	0.704		
Coronary artery disease	3.12 (0.19–51.2)	0.426		
Congestive heart failure	2.41 (1.00–5.79)	0.050		
ABO incompatibility	2.28 (1.02–5.12)	0.046	2.20 (0.97–4.96)	0.059
Hemoglobin	0.91 (0.74–1.12)	0.376		
Albumin	1.16 (0.66–2.04)	0.600		
BUN	0.97 (0.95–0.99)	0.002	0.97 (0.95–0.99)	0.005
Creatinine	0.92 (0.80–1.07)	0.274		
Sodium	1.10 (0.97–1.23)	0.132		
Potassium	0.55 (0.33–0.93)	0.025		
eGFR	1.08 (0.99–1.18)	0.077		
Beta blocker	1.88 (0.88–4.02)	0.103		
BMI of donor	1.08 (0.97–1.20)	0.147		
Operation time	1.01 (1.00–1.01)	0.008	1.01 (1.00–1.01)	0.007
Cold ischemia time	1.01 (1.00–1.02)	0.036		
Rewarming time	1.01 (0.99–1.04)	0.305		
Inotropes	2.63 (0.67–10.4)	0.168		
Vasodilator	2.89 (1.24–6.74)	0.014	2.41 (1.05–5.55)	0.038
Transfusion (RBC)	1.53 (0.57–4.11)	0.400		
ΔHR, mean	0.94 (0.63–1.39)	0.755		
ΔHR, SD	0.93 (0.75–1.16)	0.521		
ΔSBP, mean	1.18 (1.01–1.38)	0.044		
ΔSBP, SD	1.11 (1.00–1.24)	0.043		
ΔDBP, mean	1.26 (0.99–1.60)	0.058		
ΔDBP, SD	1.07 (0.98–1.16)	0.134		
ΔMBP, mean	1.23 (1.01–1.50)	0.037		
ΔMBP, SD	1.09 (0.99–1.19)	0.070		

OR, odds ratio; BMI, body mass index; HTN, hypertension; DM, diabetes mellitus; BUN, blood urea nitrogen; eGFR, estimated glomerular filtration rate; RBC, red blood cell; HR, heart rate; SD, standard deviation; SBP, systolic blood pressure; DBP, diastolic blood pressure; MBP, mean blood pressure.

**Table 3:** Clinical outcomes of recipients based on RAKT score risk assessment in robot-assisted kidney transplantation.

Outcomes	Score ≤ 7	7 < Score ≤ 9	9 < Score	*p*-Value
	(N = 52)	(N = 46)	(N = 48)	
Postoperative length of stay, days	6.2 ± 4.3	7.2 ± 3.8	11.9 ± 24.0	<0.001
eGFR at postoperative day 7, mL/min/1.73 m^2^	75.8 ± 21.0	67.7 ± 25.0	61.4 ± 23.8	0.009
eGFR at discharge, mL/min/1.73 m^2^	75.5 ± 19.0	67.0 ± 25.3	62.7 ± 22.5	0.015
Creatinine at discharge, mg/dL	1.08 ± 0.37	1.44 ± 1.19	1.54 ± 1.26	0.024
Postop. DGF, *n* (%)	0 (0.0)	2 (4.3)	1 (2.1)	0.318
MACE, *n* (%)	0 (0.0)	0 (0.0)	1 (2.1)	0.350
Rejection, *n* (%)	1 (1.9)	2 (4.3)	0 (0.0)	0.331
Readmission within 90 days, *n* (%)	11 (21.2)	7 (15.2)	13(27.1)	0.372
Postop. ICU admission, *n* (%)	0 (0.0)	2 (4.5)	8 (17.0)	0.003
Overall mortality, *n* (%)	0 (0.0)	0 (0.0)	0 (0.0)	

RAKT, robot-assisted kidney transplantation; eGFR, estimated glomerular filtration rate; DGF, delayed graft failure; MACE, major adverse cardiovascular event; ICU, intensive care unit.

## Data Availability

The datasets generated during and/or analyzed during the current study are available from the corresponding author on reasonable request.

## Supplementary Materials

The following supporting information can be downloaded at: https://www.mdpi.com/article/10.3390/medicina60081255/s1, Table S1: Scoring system for RAKT score; Figure S1: Nomogram for RAKT scoring system to predict delayed discharge after robotic kidney transplantation; Figure S2: Calibration curve for prediction model of delayed discharge after robotic kidney transplantation using new RAKT scoring system.
