# Supplementary material for: Predicting Delayed Postoperative Length of Stay Following Robotic Kidney Transplantation: Development and Simulation of Perioperative Risk Factors
Source: Medicina (Kaunas). 2024 Aug 1;60(8):1255. doi: 10.3390/medicina60081255 (PMC11356542; doi:10.3390/medicina60081255)
Supplement: Supplementary file 1 [file medicina-60-01255-s001.zip › medicina-3113323-supplementary.pdf]

Supplementary Material

Predicting Delayed Postoperative Length of Stay Following Robotic Kidney Transplantation: Development and Simulation of Perioperative Risk Factors

Sang-Wook Lee, MD PhD[1†], Kyoung-Sun Kim, MD[1†], Sung-Hoon Kim, MD PhD[1], Ji-Yeon Sim, MD PhD[1*]

Contents

Table S1. Scoring system for RAKT score

Figure S1. Nomogram for RAKT scoring system to predict delayed discharge after robotic kidney transplantation

Figure S2. Calibration curve for prediction model of delayed discharge after robotic kidney transplantation using new RAKT scoring system

Table S1. Scoring system for RAKT score

ABOi (β_{ABOi}=0.786)	Score	BUN (β_{BUN}=-0.029)	Score	Operation time ($\beta_{Op\ time}$=0.008)	Score	Vasodilator ($\beta_{Vasodilator}$=0.881)	Score
No	0	20	8.0	250	0	No	0
Yes	2.0	30	7.3	300	1	Yes	2.2
		40	6.6	350	2		
		50	5.8	400	3		
		60	5.1	450	4		
		70	4.4	500	5		
		80	3.7	550	6		
		90	2.9	600	7		
		100	2.2	650	8		
		110	1.5	700	9		
		120	0.7	750	10		
		130	0				

$$\textbf{RAKT score} = 2.0 \times \textbf{ABOi} + \frac{(130-BUN)\times 8.0}{110} + \frac{(Operation\ time-250)\times 10}{500} + 2.2 \times \textbf{Vasodilator}$$

RAKT score	Probability of delayed discharge (p)
4.4	0.05
6.3	0.1
8.3	0.2
9.7	0.3
10.8	0.4
11.8	0.5
12.8	0.6
13.9	0.7
15.3	0.8

Linear predictor = $(\textbf{RAKT score} - 11.8) \times 0.4$

Probability of delayed discharge (p) = $\frac{1}{(1+e^{(-Linear\ predictor)})}$

Linear predictor units per point : 0.4

Figure S1. Nomogram for RAKT scoring system to predict delayed discharge after robotic kidney transplantation

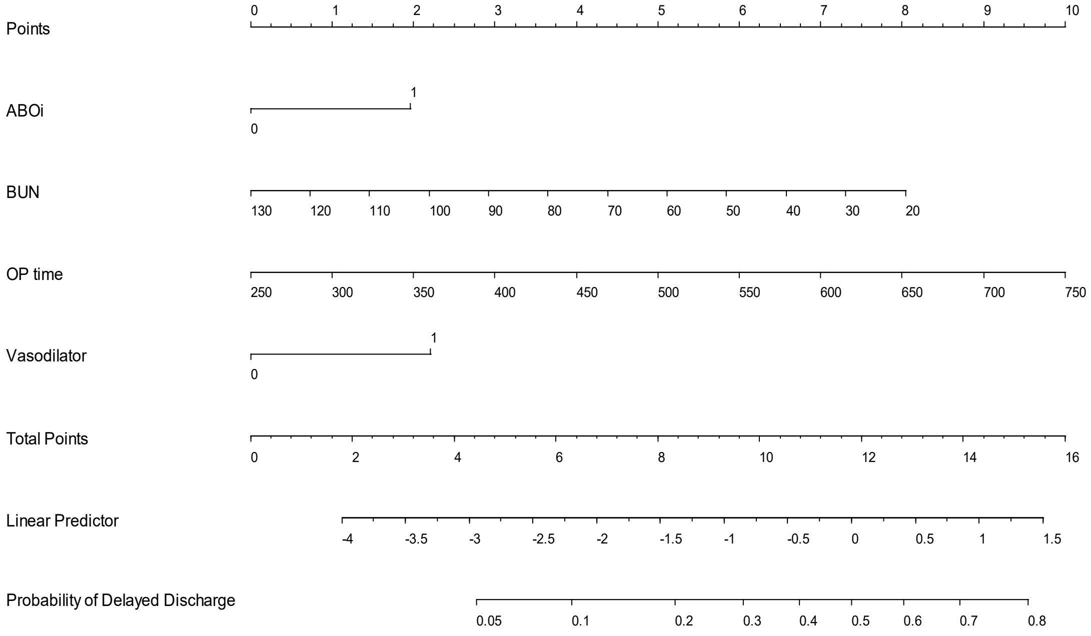

Figure S2. Calibration curve for prediction model of delayed discharge after robotic kidney transplantation using the new RAKT scoring system

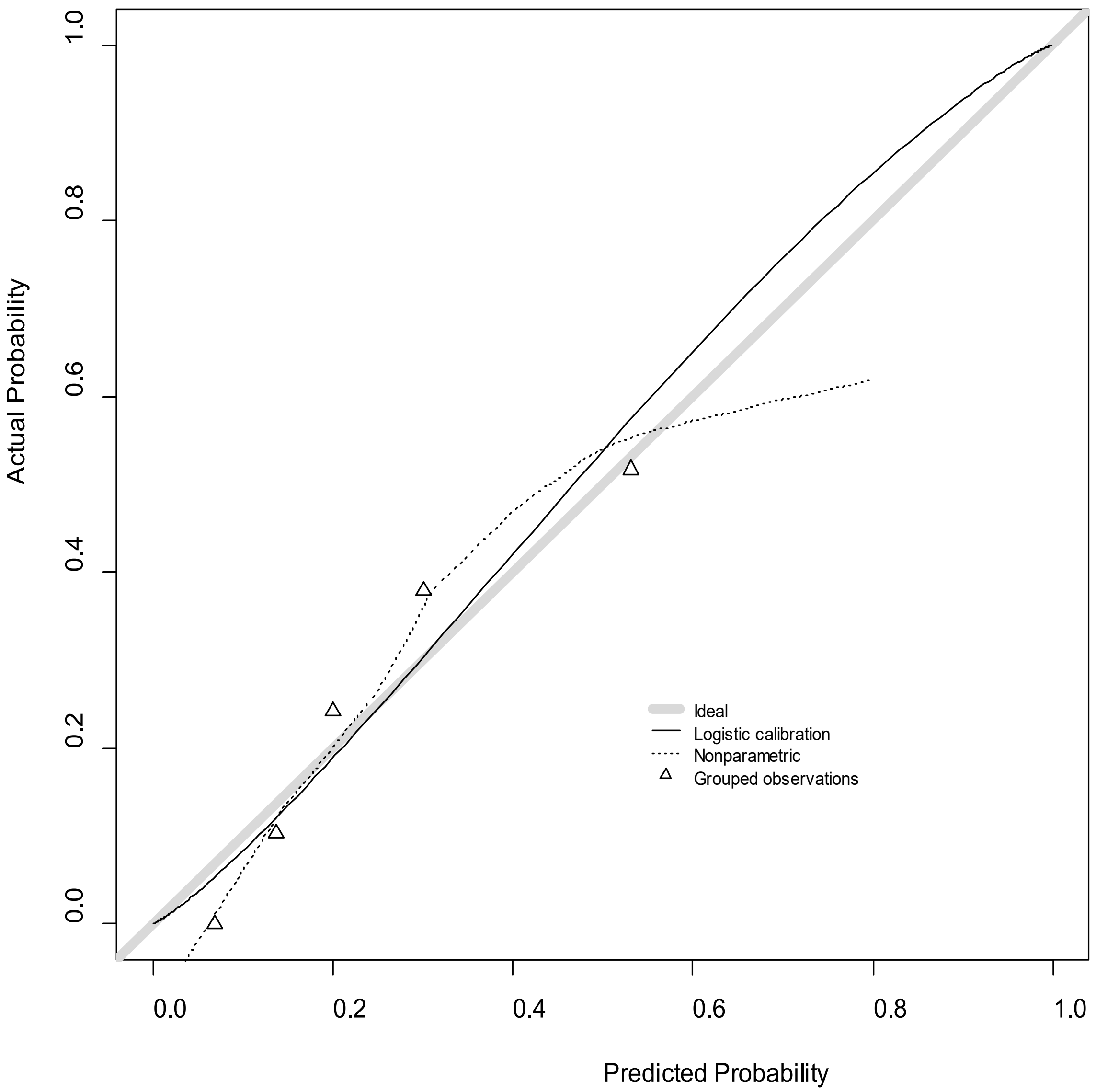

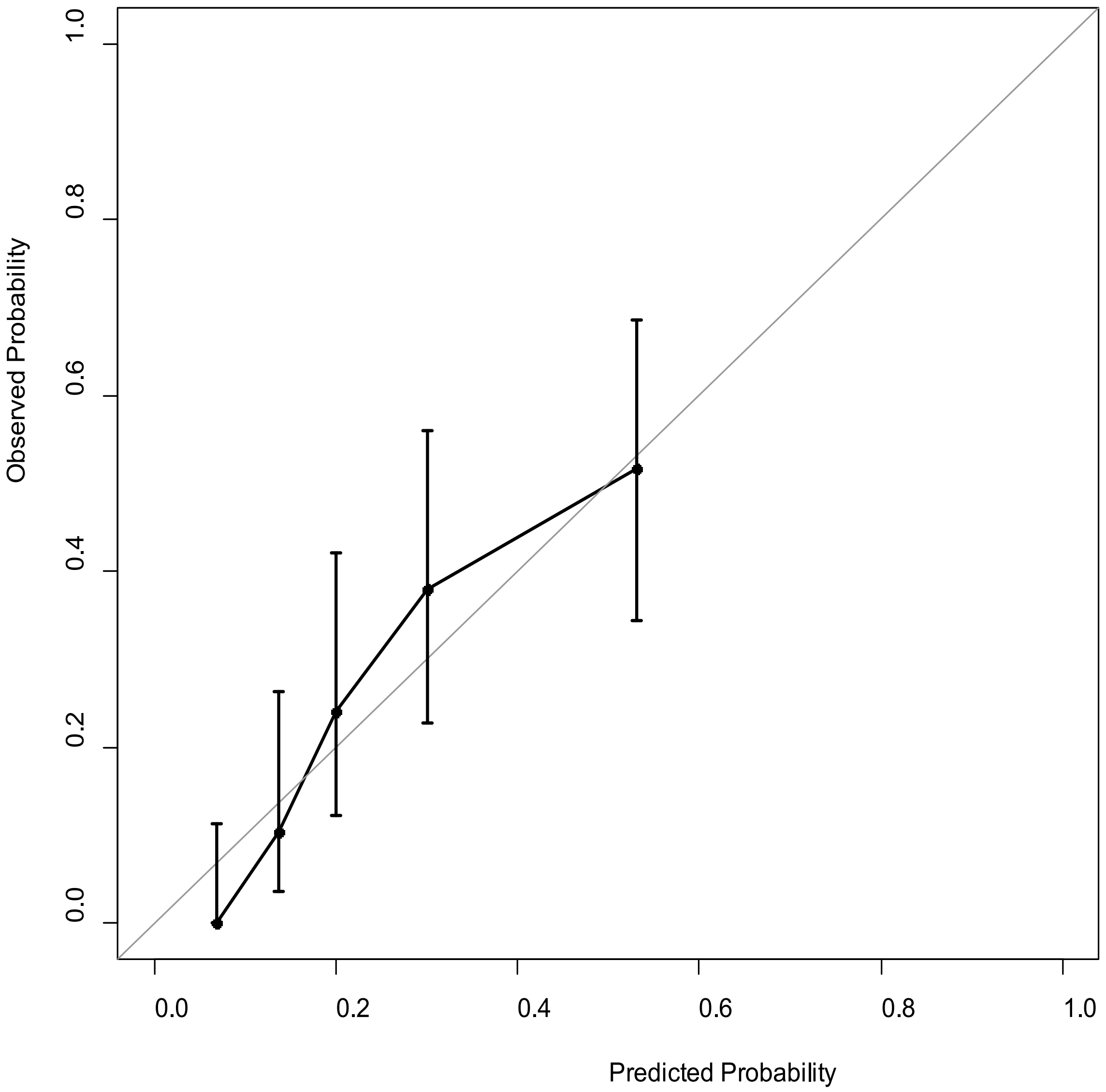
Observed Probability
0.0
0.2
0.4
0.6
0.8
1.0
Predicted Probability
